# Protein lysine acetylation regulates oral microorganisms

**DOI:** 10.3389/fcimb.2025.1594947

**Published:** 2025-05-15

**Authors:** Yuanchao Yang, Hailun He, Bingshi Liu, Zhuoyue Li, Jiaman Sun, Zhili Zhao, Yan Yang

**Affiliations:** ^1^ Xiangya Stomatological Hospital and Xiangya School of Stomatology, Central South University, Changsha, China; ^2^ Hunan Key Laboratory of Oral Health Research, Central South University, Changsha, China; ^3^ School of Life Sciences, Central South University, Changsha, China; ^4^ Department of Oral and Maxillofacial Surgery, The Second Xiangya Hospital, Central South University, Changsha, China

**Keywords:** lysine acetylation, oral microorganisms, bacterial virulence, environmental adaptation, immune escape

## Abstract

Post-translational modifications (PTMs) are integral to the regulation of protein function, stability, and cellular processes. Lysine acetylation, a widespread PTM, has been extensively characterized for its role in eukaryotic cellular functions, particularly in metabolism, gene expression, and disease progression. However, its involvement in oral microbiota remains inadequately explored. This review examines the emerging significance of lysine acetylation in modulating oral microbial communities. The oral cavity, characterized by its unique anatomical and environmental conditions, serves as a dynamic habitat where microbiota interact with host factors such as diet, immune response, pH, and the level of oxygen. Lysine acetylation enables bacterial adaptation to these fluctuating conditions, influencing microbial metabolism, virulence, and stress responses. For example, acetylation of lactate dehydrogenase in *Streptococcus mutans* reduces its acidogenicity and aciduricity, which decreases its cariogenic potential. In diverse environmental conditions, including hypoxic or anaerobic environments, acetylation regulates energy utilization pathways and enzyme activities, supporting bacterial survival and adaptation. Additionally, acetylation controls the production of extracellular polysaccharides (EPS), which are essential for biofilm formation and bacterial colonization. The acetylation of virulence factors can modulate the pathogenic potential of oral bacteria, either enhancing or inhibiting their activity depending on the specific context and regulatory mechanisms involved. This review also explores the interactions between acetylation and other PTMs, highlighting their synergistic or antagonistic effects on protein function. A deeper understanding of lysine acetylation mechanisms in oral microbiota could provide valuable insights into microbial adaptation and pathogenesis, revealing potential therapeutic targets for oral diseases.

## 1 Introduction

Post-translational modification (PTM) refers to the chemical modification of proteins following translation, which influences their structure and function. These modifications can impact protein stability, affinity, activity, and subcellular localization, thereby regulating their biological roles. In the context of oral microbiota, PTMs are integral in modulating protein synthesis, metabolism, and virulence. Oral microorganisms utilize PTMs to adapt to external stimuli and control various physiological processes. To date, over 200 distinct PTMs have been cataloged (Minguez et al., 2012), including both minor chemical modifications (e.g., phosphorylation and acetylation) and the addition of complete proteins (e.g., ubiquitination). The most common PTMs observed in oral microbiota include phosphorylation, acetylation, methylation, glycosylation, sumoylation, and lactylation (Wu et al., 2019; Zeng et al., 2020; Ma et al., 2021b).

Lysine is an amphipathic residue, characterized by a hydrophobic side chain and a positively charged ϵN-group at physiological pH. The ϵN-group in the active or binding site of proteins typically engages in salt bridge formation (Moreira et al., 2007). Acylation of lysine neutralizes the amino group’s positive charge, potentially altering the protein’s conformation. Various acylation modifications have been identified, including acetylation (Wang et al., 2010), malonylation (Peng et al., 2011), crotonylation (Montellier et al., 2012), propionylation and butylation (Chen et al., 2007), and succinylation (Lin et al., 2012). These modifications utilize metabolic intermediates as sensors to regulate metabolism and other processes, thereby coordinating metabolic pathways and signal transduction (Wellen and Thompson, 2012).

The most extensively studied lysine modification is acetylation, which occurs through reversible catalysis by protein acetyltransferases and deacetylases. Active acetyl derivatives, such as acetyl phosphate, acetyl CoA, and acetyladenylic acid, are also known to drive protein acetylation (Ramponi et al., 1975; Wagner and Payne, 2013; Weinert et al., 2013; Kuhn et al., 2014). Initially observed in histones (Allfrey et al., 1964), lysine acetylation has since been identified in various eukaryotic non-histone proteins involved in cellular metabolism, the cell cycle, aging, growth, angiogenesis, and oncogenesis (Chuang et al., 2010; Wang et al., 2010; Zhao et al., 2010; Lu et al., 2011; Carafa et al., 2012; Lin et al., 2012). In contrast, research on prokaryotic acetylation remains limited, primarily focusing on a few microbial species. Although lysine acetylation is increasingly recognized as a significant post-translational modification in bacteria, its specific roles and regulatory mechanisms within oral microbiota remain underexplored.

The oral microenvironment is intricate, shaped by the unique anatomy of the oral cavity. Oral microorganisms are highly sensitive to host factors, including diet, immune status, pH, oral hygiene, oxygen levels, and lifestyle choices (Yan et al., 2023). In contrast to slower regulatory mechanisms such as gene expression and protein turnover, lysine acetylation enables bacteria to rapidly adjust their physiological state, offering a mechanism to respond swiftly to environmental changes. For instance, under hypoxic or anaerobic conditions, lysine acetylation modulates bacterial metabolic pathways, optimizing energy utilization and regulating enzymatic activity to mitigate external stressors (Kim et al., 2015; Martín et al., 2021; Toplak et al., 2022; Ma et al., 2024a).

In addition, protein lysine acetylation is a key regulator of microbial virulence. One noteworthy illustration is the production of extracellular polysaccharides (EPS) by oral bacteria via glucosyltransferases. EPS constitutes the primary component of dental plaque biofilms, promoting bacterial colonization and aggregation. Notably, the acetylation status of glucosyltransferase correlates with its enzymatic activity (Ma et al., 2021a). Beyond local effects, oral pathogens can release virulence factors into the bloodstream, leading to systemic infections. Acetylation plays a critical role in the onset and progression of these virulence factors (Ren et al., 2017).

Protein acetylation differs from mRNA or protein synthesis in that it is not templated, instead depending on the recognition and modification by specific enzymes. This process is typically reversible and dynamic, with chemical groups being added or removed from the polypeptide chain by specialized enzymes. Beyond enzymes, certain chemical compounds also contribute to protein acetylation. For instance, acetyl coenzyme A (Ac-CoA) directly enhances the acetylation of RprY in *Porphyromonas gingivalis* (Wagner and Payne, 2013). Proteins often undergo multiple modifications, with some residues experiencing several modifications simultaneously. For example, in *Porphyromonas gingivalis*, acetylation and succinylation frequently overlap, and RprY can concurrently undergo both acetylation and phosphorylation (Li et al., 2018). The interplay between acetylation and other PTMs can produce complementary or antagonistic effects, resulting in intricate combinations that influence the structure and function of target proteins, highlighting the complexity and adaptability of regulatory mechanisms.

**Figure 1:**
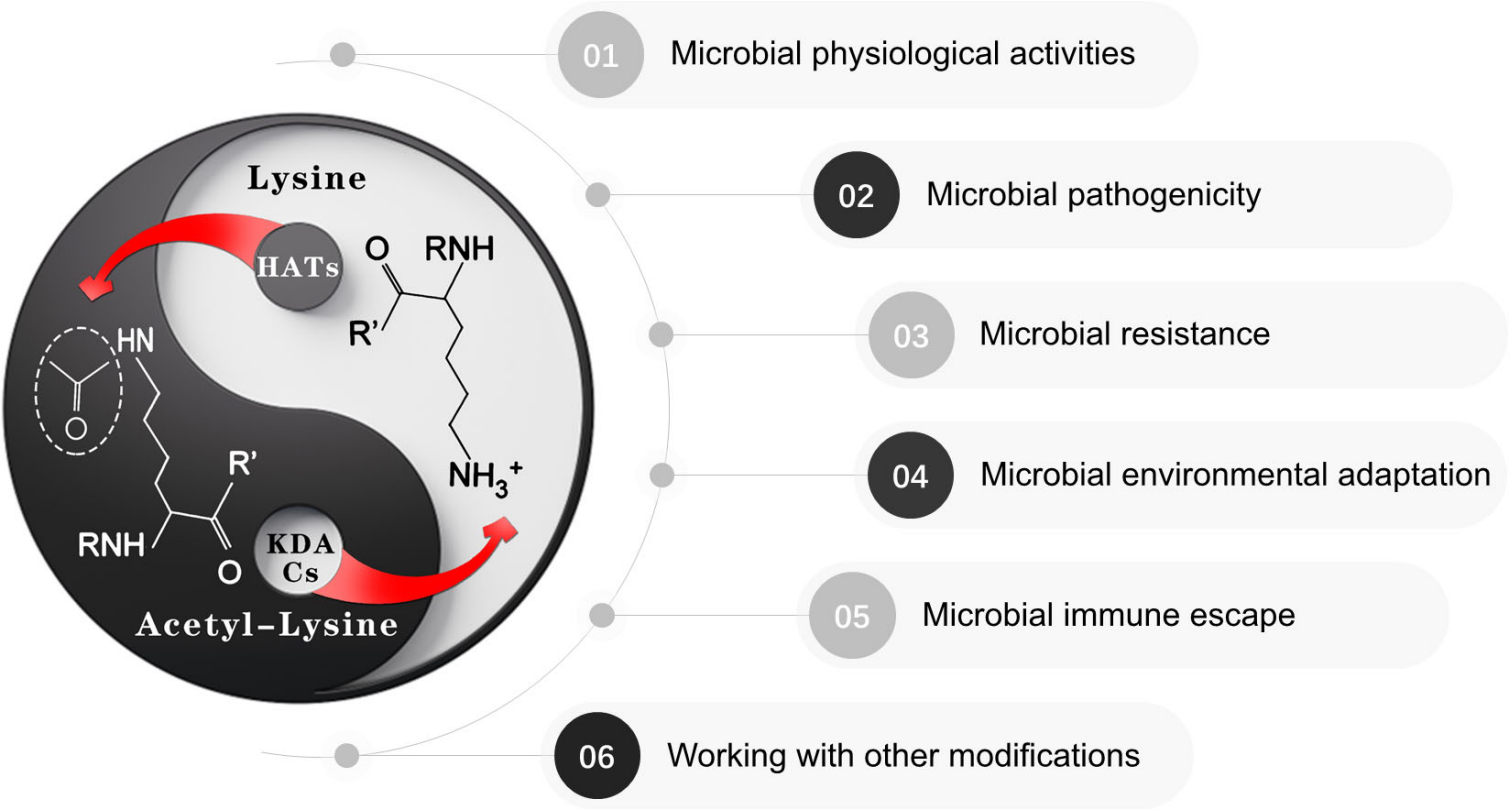
Protein lysine acetylation in oral microbiology. Oral microbiota employs dynamic acetylation/deacetylation modification mechanisms to precisely modulate critical physiological processes including physiological activities, microbial pathogenicity, drug resistance, environmental adaption, immune escape and interaction with other mechanisms. Notably, this dynamically balanced acetylation system endows pathogens with remarkable plasticity, enabling rapid epigenetic-level responses to adaptively transition between host microenvironmental conditions and external stressors. The bidirectional equilibrium inherent in this regulatory network mirrors the Taiji (Yin-Yang) philosophy in Chinese traditional thought – a cyclical interplay of complementary forces that maintains the homeostasis of living systems through dynamic equilibrium.

Recent advances in enrichment strategies for protein lysine acetylation sites have provided substantial evidence supporting the critical role of protein acetylation in regulating both the physiological and pathological processes of oral microorganisms (**Figure 1**). This review explores the significance and diverse functions of protein lysine acetylation in oral microorganisms, aiming to fill current knowledge gaps and explore the potential of acetylation as a therapeutic target for oral diseases by integrating emerging evidence across major oral microorganisms, including bacteria (e.g., *Streptococcus mutans*, *Porphyromonas gingivalis*) and fungi (e.g., *Candida albicans*), and highlighting its interplay with other post-translational modifications.

## 2 Protein acetylation

### 2.1 Protein acetylation in oral microorganisms

Lysine acetylation is regulated by two distinct mechanisms: enzymatic and non-enzymatic. The enzymatic process involves the transfer of an acetyl group from acetyl-CoA to the ϵ-amino group of deprotonated lysine, a reaction catalyzed by lysine acetyltransferases (KATs). These enzymes are classified into three primary families: (i) the Gcn5-related N-acetyltransferase (GNAT) family, named after the yeast Gcn5 protein, (ii) the MYST family, which includes human MOZ, yeast Ybf2/Sas3, yeast Sas2, and human Tip60, and (iii) the p300/CBP family, named after human p300 and CBP. While the MYST and p300/CBP families are exclusive to eukaryotic cells, the GNAT family is conserved across all domains of life (Starai and Escalante-Semerena, 2004; Chen et al., 2007; Wang et al., 2010). Several KATs have been identified in various microbial species, all belonging to the GNAT family (Hentchel and Escalante-Semerena, 2015), such as ActA and ActG in *Streptococcus mutans* (Ma et al., 2021a, 2024a) and VimA and its homolog PG1842 in *Porphyromonas gingivalis* (Mishra et al., 2018), Among them, the *Salmonella enterica* protein acetyltransferase Pat (also referred to as YfiQ *Escherichia coli*) was the first to be identified and remains the most extensively studied (Starai and Escalante-Semerena, 2004).

A non-enzymatic mechanism was identified in *Escherichia coli*, where acetyl phosphate (AcP) directly transfers its acetyl group to the ϵ-amino group of deprotonated lysine (Weinert et al., 2013; Kuhn et al., 2014), this mechanism may similarly support rapid metabolic adaptation of oral bacteria during nutritional fluctuations or stress conditions which are common in the oral cavity (Di et al., 2023). AcP, a high-energy intermediate in the phosphotransacetylase-acetate kinase (Pta-AckA) pathway, is traditionally recognized as a donor of phosphorylation groups for specific response regulators within two-component signaling systems (Lukat et al., 1992). The *yfiQ* deletion mutant shows minimal impact on global acetylation levels, while increasing AcP concentrations correspond to elevated global acetylation, indicating that AcP-dependent acetylation is less specific than its enzymatic counterpart and may significantly influence bacterial physiological processes.

### 2.2 Protein deacetylation in oral microorganisms

Lysine deacetylases (KDACs) enzymatically remove acetyl groups. Currently, two primary KDAC families have been identified and categorized into four groups: the NAD+-dependent sirtuin family (class III) (Blander and Guarente, 2004) and the zinc-dependent Rpd3/Hda1 family (classes I, II, and IV) (Yang and Seto, 2008).

The NAD+-dependent CobB, the most extensively studied bacterial sirtuin, was initially characterized in *Streptococcus faecalis* (Starai et al., 2002). Its homologs have since been identified in other bacteria, including *Escherichia coli* (Zhao et al., 2004)and *Streptomyces* (Mikulik et al., 2012). CobB typically does not exhibit a preference for Pat-dependent or AcP-dependent acetylated lysine, with its substrates participating in diverse cellular processes (AbouElfetouh et al., 2015). For instance, CobB deacetylates both Pat- and AcP-dependent acetylated DnaA (Zhang et al., 2016). Initially regarded as the sole histone deacetylase (HDAC) in *Escherichia coli* due to the absence of other HDAC homologs, CobB’s role was reassessed upon the identification of a novel deacetylase, YcgC, in *Escherichia coli* (Tu et al., 2015). YcgC, a member of the serine hydrolase family, lacks significant homology with known KDACs and is neither NAD+- nor Zn2+-dependent. It catalyzes substrate deacetylation through mechanisms distinct from established deacetylases, targeting a different set of acetylated proteins than those regulated by CobB in *Escherichia coli*. Thus, YcgC and its homologs may constitute a novel bacterial deacetylase family. Additionally, MSMEG_4620, a SIRT4 homolog in Mycobacterium tuberculosis, exhibits both deacetylase and auto-ADP-ribosyltransferase activities (Tan et al., 2016). Consequently, further investigation into novel microbial deacetylases and the interplay between acetylation factors and deacetylases will provide valuable insights.

## 3 Regulation of oral bacteria by protein acetylation

### 3.1 Streptococcus mutans

In *Streptococcus mutans*, lysine acetylation, a widespread and dynamic PTM, is integral to bacterial metabolic regulation and pathogenicity. It has been demonstrated that *Streptococcus mutans* utilizes sucrose to synthesize exopolysaccharide via glucosyltransferases (Gtfs), a process regulated by lysine acetylation. Analysis of protein acetylation dynamics revealed that 22.7% of proteins were acetylated, with significant enrichment in glycolysis/gluconeogenesis and RNA degradation pathways (Lei et al., 2021).

Acetyltransferase ActG has been shown to acetylate GtfB and GtfC, inhibiting their activity and thus reducing EPS synthesis and biofilm formation. However, site-directed mutagenesis of specific lysine residues was not performed to pinpoint the functional acetylation sites (Ma et al., 2021a). Furthermore, acetylation of lactate dehydrogenase (LDH) also inhibits its enzymatic activity, decreasing the cariogenic potential of *Streptococcus mutans* (Ma et al., 2022). ActA, a member of the GNAT family in *Streptococcus mutans*, regulates bacterial adaptation and competitiveness against oxidative stress through acetylation of LDH and pyruvate kinase (PykF) (Ma et al., 2024a). In contrast, the NAD^+^-dependent deacetylase YkuR reverses Gtfs acetylation and restores their enzymatic activity, thereby promoting EPS production and biofilm formation. Deletion of ykuR elevates global acetylation and attenuates cariogenicity *in vivo* (Ma et al., 2024b).

Aspirin, a non-enzymatic acetylating agent, inhibits the growth and EPS production of *Streptococcus mutans* and reduces the enzymatic activity of Gtfs (Lin et al., 2024), further supporting the role of protein acetylation in antimicrobial and anti-biofilm applications.

Additionally, small RNA (sRNA) interacts with lysine acetylation in *Streptococcus mutans*. SmsR1 sRNA modulates protein acetylation levels and LDH activity by regulating the concentration of the *pdhC* gene and its metabolite acetyl-CoA (Li et al., 2024), reflecting bacterial adaptability to environmental stress.

These findings highlight the complex regulatory role of lysine acetylation in *Streptococcus mutans*, influencing bacterial virulence (e.g., biofilm formation) and pathogenicity (e.g., cariogenicity, environmental adaptation) (**Table 1**), and provide a theoretical foundation for developing new strategies to prevent and treat dental caries.

**Table 1:** Lysine acetylation in *Streptococcus mutans*.

Lysine acetylation in *Streptococcus mutans*			
Mechanism	Effect	Biological function	References
Acetylation of GtfB and GtfC by ActG	Inhibits the enzymatic activity of GtfB and GtfC, reducing EPS synthesis and biofilm formation	Inhibits biofilm formation	Ma et al., 2021a
Acetylation of LDH	Inhibits LDH activity, reducing acidogenic potential	Decreases *Streptococcus mutans*’ cariogenic potential	Ma et al., 2022
Acetylation of LDH and PykF by ActA	Inhibits bacterial adaptation to oxidative stress	Inhibits oxidative stress tolerance and competitiveness of *Streptococcus mutans*	Ma et al., 2024a
YkuR-mediated NAD^+^-dependent deacetylation of Gtfs	Restores Gtf activity and increases EPS synthesis	Enhances *Streptococcus mutans* biofilm formation and virulence	Ma et al., 2024b
Non-enzymatic acetylation by ASA	Inhibits bacterial growth, reduces EPS production and Gtfs enzyme activity	Provides antimicrobial and anti-biofilm effects by targeting acetylation	Lin et al., 2024
Interaction of SmsR1 sRNA with lysine acetylation	Modulates acetylation levels and LDH activity by regulating *pdhC* and acetyl-CoA	Environmental stress response and metabolic flexibility	Li et al., 2024

### 3.2 Porphyromonas gingivalis

*Porphyromonas gingivalis*, a Gram-negative anaerobic bacterium, is a key pathogen in chronic periodontitis. Through dynamic analysis of protein acetylation, 130 lysine acetylation sites from 92 *Porphyromonas gingivalis* proteins were identified, with the majority associated with 45 metabolically active proteins. These proteins are involved in multiple metabolic pathways, where enzymes catalyzing consecutive reactions within the same pathway are frequently acetylated. Notably, 12 enzymes in the anaerobic amino acid fermentation pathway, critical for energy production, also undergo lysine acetylation. This indicates that lysine acetylation plays a central role in the metabolic regulation of *Porphyromonas gingivalis*, contributing significantly to its survival and metabolic adaptation during infection (Butler et al., 2015).

VimA, a multifunctional protein in *Porphyromonas gingivalis*, regulates critical biosynthetic pathways through its acetyltransferase activity. It plays a role in the glycosylation and anchoring of bacterial surface proteins, lipid A synthesis, and the maintenance of oxidative stress tolerance. Notably, VimA and its homolog PG1842 acetylate the gingipain precursor pro-RgpB at key lysine residues (Y230, K247, and K248), facilitating its activation and maturation, which enhances both the invasive and biofilm-forming capacities of *Porphyromonas gingivalis* (Mishra et al., 2018). *VimA*-deficient mutants, such as FLL92, exhibit reduced invasion efficiency into host cells; however, supplementation with VimA restores invasive potential. This highlights VimA’s essential role in modulating the pathogenicity of *Porphyromonas gingivalis*. Additionally, VimA influences branched-chain amino acid metabolism by regulating acetyl-CoA levels, thereby affecting lipid A biosynthesis (Aruni et al., 2012). Lipid A, a major component of the outer membrane of *Porphyromonas gingivalis*, is crucial for immune evasion. Studies have shown that *VimA*-deficient mutants display impaired survival under oxidative stress, suggesting that VimA contributes to bacterial survival in fluctuating oral environments by enhancing membrane stability and stress tolerance.

Protein acetylation is integral to the transcriptional regulation of *Porphyromonas gingivalis*. Acetylation of the transcription factor RprY impairs its DNA-binding ability, thereby diminishing its transcriptional activation of target genes (Li et al., 2018).

A significant aspect of lysine acetylation in *Porphyromonas gingivalis* proteins is its interaction with lysine succinylation (Ksuc). In *Porphyromonas gingivalis* ATCC 33277, a substantial overlap between Ksuc and Kac occurs, particularly in ribosomal and metabolic proteins, reflecting the complexity of PTMs in bacterial physiological regulation (Zeng et al., 2020).

In conclusion, protein acetylation is central to the physiological functions, metabolic regulation, and pathogenicity of *Porphyromonas gingivalis*. By modulating various metabolic pathways, protein sorting, and surface structure synthesis, acetylation enhances the bacterium’s environmental adaptability, invasiveness, and biofilm formation capabilities (**Table 2**). These insights present promising molecular targets for the development of novel antimicrobial therapies.

**Table 2:** Lysine acetylation in *Porphyromonas gingivalis*.

Lysine acetylation in *Porphyromonas gingivalis*			
Mechanism	Effect	Biological function	References
VimA-mediated acetylation of pro-RgpB	VimA and its homolog PG1842 acetylate the gingipain precursor pro-RgpB at key lysine residues, facilitating its activation and maturation	Enhances bacterial invasiveness and biofilm-forming capacity	Mishra et al., 2018
VimA regulation of branched-chain amino acid metabolism	VimA indirectly affects Lipid A synthesis by modulating acetyl-CoA levels	Maintains membrane stability, enhances oxidative stress tolerance, and promotes immune evasion	Aruni et al., 2012
Acetylation of the transcription factor RprY	Acetylation of RprY reduces its DNA-binding ability	Weakens the transcriptional activation of target genes and participates in transcriptional regulation	Li et al., 2018
Crosstalk between acetylation and succinylation	There is a significant overlap between Kac (acetylation) and Ksuc (succinylation) modifications in ribosomal and metabolic proteins	Forms a complex network of post-translational regulation, modulating bacterial physiology	Zeng et al., 2020

### 3.3 Actinomycetes

Protein lysine acetylation, a critical PTM, is integral to the regulation of various biological processes in Actinomycetes. In fact, “Amino acid sensing” was specifically observed only in Actinomycetes, based on recent studies identifying ACT domain-containing GNATs (Lu et al., 2017; Lammers, 2021). However, similar to other organisms, such as *Streptococcus mutans* and *Porphyromonas gingivalis*, akin regulatory mechanisms such as enzyme activity modulation and metabolic network regulation via acetylation are also present in Actinomycetes (Hesketh et al., 2002).

#### 3.3.1 Amino acid sensing: GNATs and functional properties of the ACT domain

Recent studies have identified a distinct class of GNATs in Actinomycetes, which are integral to acetylation reactions via their unique domains and regulatory mechanisms. Specifically, these enzymes feature two functional domains: the ACT (amino acid binding) domain and the GNAT (N-acetyltransferase) domain. The ACT domain detects specific amino acids, thereby allosterically modulating the acetyltransferase activity of the GNAT domain (Lu et al., 2017). This “amino acid-induced allosteric regulation” implies that amino acids influence acetyltransferase catalytic activity by binding to the ACT domain. GCN5-like acetyltransferases can be classified into two groups based on the type of amino acids induction: Asn-activated PatA and Cys-activated PatA. The former is predominantly found in *Streptomyces*, while the latter is more widespread across other actinomycete species (Lu et al., 2017). This distinction highlights that acetyltransferase regulation not only fine-tunes metabolic processes through amino acid sensing but also correlates with the physiological traits and ecological adaptability of different actinomycete species.

#### 3.3.2 Enzyme activity regulation: dynamic balance between acetyltransferases and deacetylases

In Actinomycetes, the balance between acetyltransferases (e.g., AcuA) (Gardner et al., 2006), and deacetylases (e.g., Sirtuins) (Tucker and Escalante-Semerena, 2013) plays a pivotal role in fine-tuning enzyme activity, particularly under energy stress or nutrient-limited conditions. This dynamic regulation is critical for adapting metabolic fluxes and optimizing secondary metabolite biosynthesis.Acetylation levels also influence the synthesis of secondary metabolites by modulating the activity of specific transcription factors or metabolic enzymes. By altering transcription factor binding affinity to gene promoters, acetylation can either enhance or suppress DNA binding, thereby regulating the expression of genes involved in secondary metabolite production. This regulatory mechanism is essential for coordinating the biosynthesis of compounds such as antibiotics (e.g., streptomycin) and non-ribosomal peptides in response to environmental or metabolic signals (Martín et al., 2021).

Additionally, acetylation of key signaling enzymes or synthetases, such as acetyl-CoA synthetase (Tucker and Escalante-Semerena, 2013), can alter their catalytic efficiency and affect metabolic flux. This modification not only regulates enzyme activity but also influences the overall metabolic pathway, promoting or inhibiting the synthesis of specific metabolites. For instance, in *Streptomyces roseosporus*, acetylation of non-ribosomal peptide synthetases suggests a significant role for acetylation in secondary metabolism (Liao et al., 2014). In *Streptomyces griseus*, acetylation of the StrM enzyme, particularly at lysine site 70, reduces its activity, thereby limiting streptomycin biosynthesis (Ishigaki et al., 2017).

#### 3.3.3 Fine regulation of metabolic networks: from amino acid metabolism to secondary metabolite biosynthesis

The coordinated regulation of amino acid sensing and enzyme activity forms a precise regulatory mechanism within the intricate metabolic network of Actinomycetes. Acetylation is not only involved in central metabolic processes but also significantly influences amino acid metabolic pathways. Studies indicate that AAPatA acetyltransferases, which utilize Asn or Cys as sensing molecules, can modulate cellular metabolic pathways by regulating enzymes involved in aspartate and cysteine metabolism (Xu et al., 2014; Lu et al., 2017).

This acetylation-driven network is particularly responsive to environmental fluctuations. Under changing nutritional conditions or stress, acetylation rapidly adjusts bacterial metabolic pathways by altering the modification states of specific transcription factors and redox enzymes, such as GrhO6 (Toplak et al., 2022). Such regulatory flexibility supports the synthesis of secondary metabolites, enabling Actinomycetes to efficiently adapt to diverse habitats and produce specialized secondary metabolites and antibiotics.

Protein acetylation regulation in Actinomycetes extends beyond traditional cellular functions, including amino acid sensing, enzyme activity modulation, and the precise regulation of metabolic networks (**Table 3**). Future research should focus on elucidating the specific roles of acetyltransferases in actinomycete antibiotic synthesis and their potential as targets for developing new biological agents or antibiotics. In-depth mechanistic studies could further uncover the complexities of microbial metabolic regulation, offering novel insights for bioengineering and the development of antimicrobial drugs.

**Table 3:** Lysine acetylation in Actinomycetes.

Lysine acetylation in Actinomycetes			
Mechanism	Effect	Biological function	References
Amino acid sensing via GNATs and ACT domains	Acetyltransferase activity modulated allosterically by amino acids binding to the ACT domain	Fine-tunes metabolic processes and correlates with ecological adaptability of Actinomycetes	Lu et al., 2017
Dynamic balance between acetyltransferases and deacetylases	Acetylation levels regulated by AcuA acetyltransferase and Sirtuins deacetylases	Modulates metabolic state in response to energy deprivation or stress conditions	Gardner et al., 2006; Tucker and Escalante-Semerena, 2013
Acetylation of transcription factors and metabolic enzymes	Acetylation alters transcription factor DNA binding, influencing secondary metabolite synthesis	Regulates biosynthesis of antibiotics and non-ribosomal peptides	Martín et al., 2021
Acetylation of acetyl-CoA synthetase	Modifies catalytic efficiency and metabolic flux	Influences overall metabolic pathways and secondary metabolite production	Tucker and Escalante-Semerena, 2013
Acetylation of non-ribosomal peptide synthetases	Modulates the activity of enzymes involved in secondary metabolism	Enhances or inhibits the synthesis of secondary metabolites such as antibiotics	Liao et al., 2014
Acetylation of StrM enzyme in *Streptomyces griseus*	Reduces StrM activity, limiting *streptomycin* biosynthesis	Regulates antibiotic production in response to metabolic signals	Ishigaki et al., 2017
Acetylation in amino acid metabolism	AAPatA acetyltransferases modulate aspartate and cysteine metabolism	Regulates cellular metabolic pathways and adapts to changing environmental conditions	Xu et al., 2014; Lu et al., 2017
Acetylation-driven metabolic network regulation	Alters transcription factors and redox regulatory enzymes to modulate bacterial metabolic pathways	Supports the synthesis of secondary metabolites and adaptation to diverse habitats	Toplak et al., 2022

## 4 Regulation of oral *Candida albicans* by protein acetylation

In recent years, the infection rate of *Candida albicans*, a common opportunistic fungal pathogen, has risen significantly. The growing prevalence of drug resistance and the limited availability of effective antifungal agents present substantial challenges to clinical management. During investigations into its pathogenic mechanisms and drug resistance, lysine acetylation has emerged as a key epigenetic modification. By regulating chromatin structure, gene expression, and signal transduction, lysine acetylation plays a critical role in the growth, virulence, morphological transformation, and stress response of *Candida albicans*. As such, it represents a promising target for the development of novel antifungal therapies.

Histone acetylation regulates key physiological processes in *Candida albicans*, such as DNA replication, transcription, and DNA repair, influencing growth, virulence, drug resistance, and environmental adaptability. Histone acetyltransferases (HATs) and histone deacetylases (HDACs) control the acetylation and deacetylation of histones, respectively. During replication, newly synthesized histones are acetylated by HATs, deposited on DNA, and later deacetylated by HDACs; histone chaperones recognize acetylation patterns during this process (Shahbazian and Grunstein, 2007). Additionally, histone acetylation alters chromatin structure, affecting the recruitment of DNA-binding proteins and transcription factors, which in turn modulates gene transcription (Shahbazian and Grunstein, 2007). For example, in *Candida albicans*, acetylation of H3K56 promotes gene transcriptional activation by increasing chromatin accessibility, with its level closely linked to transcription factor binding on chromatin (Wurtele et al., 2010). This modification creates a favorable environment for gene transcription, reshaping the transcriptional profile of *Candida albicans*, aiding host adaptation, and enhancing pathogenicity. Rtt109, another HAT, plays a critical role in DNA damage repair and pathogenicity. Deletion of the rtt109 gene results in heightened endogenous DNA damage and increased susceptibility to host macrophages (Lopes da Rosa et al., 2010). Hat1 also contributes significantly to DNA repair; its loss leads to rapid DNA damage accumulation and shifts the growth pattern from yeast to pseudohyphal form, ultimately reducing survival of *Candida albicans* (Tscherner et al., 2012). These findings emphasize the essential role of histone acetylation in the physiological and pathogenic processes of *Candida albicans*.

Histone acetylation influences the morphological plasticity of *Candida albicans*, determining its environmental adaptability and virulence. *Candida albicans* can transition between yeast, pseudohyphal, and hyphal forms, with the filamentous forms (pseudohyphae and hyphae) playing a critical role in promoting fungal infection and the formation of drug-resistant biofilms (Odds, 1985; Sudbery, 2011). Various enzymes, including HATs and HADCs, are key regulators of this morphological transition. Altered HAT expression significantly impacts *Candida albicans*’s ability to adapt to environmental changes. For example, deletion of the SWR1 gene, which is involved in H2A.Z histone variant deposition, induces chromatin structure changes, promoting the transition between white and opaque morphologies. The SWR1 complex also regulates nucleosome positioning at the WOR1 promoter, a master regulator of the white-opaque switch essential for maintaining phenotypic plasticity (Guan and Liu, 2015). Additionally, MYST family HATs, such as Esa1 and Sas2, contribute to hyphal growth. Loss of Esa1 specifically impairs hyphal formation without affecting overall growth, highlighting the significance of HATs in regulating morphology and pathogenicity. Moreover, certain HADCs, like the NuA4 complex, regulate histone acetylation via enzymes such as Yng2, further influencing morphological transitions (Wang et al., 2013). These insights emphasize the critical role of histone acetylation in controlling *Candida albicans*’s morphological plasticity and its adaptation to various host environments and pathogenic states.

Lysine acetylation is integral to *Candida albicans*’ ability to respond to host immune defenses. This pathogen evades immune detection by modulating the expression of oxidative stress response genes, a process tightly regulated by dynamic histone acetylation (Kim et al., 2018). In addition to transcriptional regulation, acetylation also influences structural adaptations that facilitate immune evasion, as the histone deacetylase Sir2 has been shown to promote systemic candidiasis by remodeling the fungal cell wall, thereby reducing the exposure of immunogenic components like mannan and β-glucan. This remodeling diminishes recognition by the host’s innate immune system and enhances fungal adhesion to host cells, contributing to increased virulence (Yang et al., 2024). Notably, lysine acetylation at specific sites, such as H3K56, influences *Candida albicans*’s capacity to tolerate oxidative stress and escape immune surveillance (Conte et al., 2024). By altering chromatin structure, histone acetylation modulates gene expression in response to stressors like reactive oxygen species generated by the host immune system. The role of HATs in this process is critical; for instance, *Candida albicans* deficient in the lysine acetyltransferase Gcn5 exhibits reduced survival in THP-1 macrophages and heightened susceptibility to various stressors (Yu et al., 2022).

Furthermore, interactions between host-derived signals and epigenetic modifications, such as lysine acetylation, influence pathogen morphology, enhancing its adaptability to the host environment. Although the molecular mechanisms underlying these processes remain under investigation, it is evident that lysine acetylation plays a key mediating role in *Candida albicans*’ resistance to host immune responses.

Lysine acetyltransferases are essential in mediating drug resistance and pathogenicity. The glucosamine-6-phosphate acetyltransferase encoded by the GNA1 gene influences the growth and virulence of *Candida albicans*, with its deletion resulting in a marked reduction in pathogenicity (Mio et al., 2000). Similarly, the HAT encoded by the NGG1 gene is involved in morphological transformation and virulence; its knockout substantially diminishes the strain’s pathogenic potential (Li et al., 2017a). Furthermore, Hsp90 plays a critical role in drug resistance and morphogenesis. Research has shown that KDACs exhibit functional redundancy in regulating Hsp90 activity, and their inhibition can enhance the effectiveness of antifungal treatments (Li et al., 2017b).

In conclusion, lysine acetylation contributes significantly to the morphological adaptability, immune evasion, and drug resistance of *Candida albicans* by modulating chromatin structure, gene expression, and stress responses (**Table 4**). These epigenetic mechanisms not only enhance understanding of *Candida albicans* pathogenicity but also open avenues for the development of novel antifungal therapies. Although direct evidence of oral-specific acetylation mechanisms is currently lacking, the unique characteristics of the oral environment and the role of acetylation in gene regulation warrant further investigation into this area.

**Table 4:** Lysine acetylation in oral *Candida albicans*.

Lysine acetylation in *oral Candida albicans*			
Mechanism	Effect	Biological function	References
Acetylation of H3K56	Promotes gene transcription by increasing chromatin accessibility	Enhances transcriptional activation, aiding host adaptation and pathogenicity	Wurtele et al., 2010
Rtt109-mediated acetylation	Plays a role in DNA damage repair and pathogenicity	Protects against DNA damage and enhances survival in macrophages	Lopes da Rosa et al., 2010
Hat1-mediated acetylation	Modifies chromatin structure and promotes DNA repair	Impacts DNA damage response and growth pattern changes, reducing survival	Tscherner et al., 2012
SWR1 complex-mediated acetylation	Modulates nucleosome positioning and chromatin structure changes	Regulates white-opaque morphological transition and phenotypic plasticity	Guan and Liu, 2015
MYST family HATs, including Esa1 and Sas2, acetylation	Impairs hyphal growth upon Esa1 deletion	Contributes to hyphal growth and virulence	Wang et al., 2013
Histone acetylation by NuA4 complex	Regulates histone acetylation, influencing morphological transitions	Affects hyphal transition and virulence	Wang et al., 2013
Gcn5-mediated acetylation in response to host signals	Alters histone acetylation, influencing survival in macrophages	Regulates stress response and immune evasion	Yu et al., 2022
Glucosamine-6-phosphate acetyltransferase by GNA1	Regulates growth and virulence	Modulates pathogenicity and immune evasion	Mio et al., 2000
NGG1 gene-encoded HAT activity	Involves in morphological transformation and virulence	Influences pathogenic potential through morphological regulation	Li et al., 2017a
Hsp90 regulation by KDACs	Enhances antifungal treatment effectiveness by regulating Hsp90 activity	Plays a critical role in drug resistance and morphogenesis	Li et al., 2017b

## 5 Potential of protein acetylation in the prevention and treatment of oral diseases

Protein lysine acetylation plays a critical role in microbial physiology, influencing both metabolism and pathogenic potential. Recently, it has garnered significant attention in the context of oral diseases. Lysine acetylation influences the onset and progression of conditions such as dental caries, periodontal disease, and oral mucosal disorders by modulating microbial growth, metabolism, and virulence, offering novel insights and therapeutic strategies for their prevention and treatment.

Histone acetyltransferase-based treatments have been utilized in various clinical applications. Many cellular proteins undergo acetylation post-translationally, resulting in alterations to their structure and function. Consequently, HDAC inhibitors have been developed as therapeutic agents, with two currently approved by the US FDA for the treatment of cutaneous T-cell lymphoma (Richon et al., 2009). Moreover, lysine acetyltransferases are emerging as promising drug targets (Folders et al., 2000).

In dental caries research, aspirin, a non-enzymatic acetylating agent, reduces Gtfs activity in *Streptococcus mutans*. It also inhibits the growth of *Streptococcus mutans* and the production of EPS, highlighting the potential of protein acetylation in anti-caries therapies (Lin et al., 2024).

In the gingival tissue of periodontitis patients, dysregulated histone acetylation and deacetylation, along with alterations in DNA methylation, are closely linked to immune and inflammatory responses. In experimental periodontitis models, histone acetylation-targeting treatments, such as HDAC inhibitors (HDACi) or acetylated histone mimetics, effectively prevent alveolar bone loss (Cantley et al., 2011; Meng et al., 2014; Li et al., 2020), offering a novel therapeutic approach for periodontal diseases.

In *Candida albicans*, lysine 56 of histone H3 undergoes acetylation by the acetyltransferase Rtt109p, and pharmacological inhibition or genetic modulation of this enzyme has been proposed as a potential antifungal therapeutic strategy. Acetylation events, often challenging to detect, can significantly impact protein functions, including stability and crystallinity (Mahon et al., 2015). For therapeutic proteins, such modifications may influence immunogenicity and biological activity, thereby affecting safety and efficacy in clinical applications (Walsh and Jefferis, 2006).

Overall, lysine acetylation plays a key role in regulating the metabolism and pathogenicity of oral pathogens by modulating the activity of critical enzymes. Research in this area not only enhances understanding of the pathogenic mechanisms of oral pathogens but also lays a foundation for the development of novel antibacterial and antifungal therapies. Targeting specific acetylases could substantially improve the efficacy of current treatments and offer potential solutions to manage drug resistance. Future investigations should focus on the role of acetylation in the oral microbiota and its impact on host-pathogen interactions, thus paving the way for new strategies in maintaining oral health and addressing oral diseases.

## 6 Conclusion and prospect

Acetylation, a key PTM, is central to the functional regulation of oral microorganisms. It modulates various bacterial physiological processes, including metabolism, cell signaling, virulence factor expression, and host-pathogen interactions, by altering protein properties such as structure, activity, localization, and interactions with other biomolecules.

This modification significantly impacts the metabolic state of oral bacteria. While acetylation is typically catalyzed by acetyltransferases, it can also occur non-enzymatically via Ac-CoA (Paik et al., 1970; Yan et al., 2008) or acetyladenylate (Ramponi et al., 1975). As a metabolic intermediate, acetylation enables bacteria to adapt to environmental fluctuations and shifts in metabolic activity, thereby playing a crucial role in the regulation of bacterial physiology and pathogenicity.

Acetylation, along with other PTMs such as succinylation and phosphorylation, collectively regulates bacterial protein functions, forming a complex modification network (Latham and Dent, 2007). This cross-regulation of multiple modifications is essential for controlling bacterial virulence factors, adaptive responses, and metabolic processes. For example, in *Porphyromonas gingivalis*, substantial overlap has been observed between acetylation and succinylation sites—especially on ribosomal and metabolic proteins—suggesting potential competitive or cooperative regulation of key cellular functions (Zeng et al., 2020). Additionally, the response regulator RprY in *Porphyromonas gingivalis* is modified by both acetylation and phosphorylation, with each modification exerting distinct effects on its DNA-binding activity and transcriptional regulation (Li et al., 2018). These findings underscore the existence of an intricate PTM crosstalk network that supports bacterial adaptation to fluctuating environmental and host-derived conditions. Nevertheless, systematic studies on the functional interplay among different PTMs in oral microorganisms remain limited. Future research should aim to elucidate the molecular mechanisms and biological consequences of these interactions—particularly the synergistic or antagonistic effects between acetylation, phosphorylation, and succinylation—in governing microbial adaptation, pathogenicity, and immune evasion.

Despite extensive research on acetylation, significant gaps remain in the study of acetylation in oral bacteria. Most investigations have concentrated on a limited number of oral pathogens, such as *Porphyromonas gingivalis* and *Streptococcus mutans*. It is important to note that lysine acetylation is a widely conserved modification across diverse oral microbial taxa. Nevertheless, functional studies and comprehensive acetylomic profiling in other clinically relevant oral species—such as *Fusobacterium nucleatum*, *Prevotella intermedia*, and *Veillonella* spp.—remain limited. To address this, future research should broaden the scope of study to include a wider range of oral bacteria and conduct comprehensive acetylomics analyses to better elucidate the acetylation patterns and functions across diverse physiological and pathological states.

From a clinical standpoint, acetylation represents a key mechanism in regulating bacterial virulence and drug resistance, offering potential new targets for the treatment of oral infections. Investigating the role of acetylation in oral microbiota could provide a theoretical foundation for the development of novel antibacterial strategies. Future research should not only examine the fundamental role of acetylation in bacterial function but also explore strategies to mitigate bacterial pathogenicity through acetylation regulation, particularly by targeting the acetylation of key virulence factors to enhance therapeutic outcomes.

In conclusion, acetylation plays a crucial role in the adaptation, virulence, and immune evasion of oral microorganisms. Advances in technologies such as metabolomics, proteomics, and mass spectrometry will further elucidate the role of acetylation in bacterial physiological and pathological processes. This enhanced understanding will not only clarify the mechanisms underlying the virulence of oral bacteria but also open new avenues for the treatment of oral diseases.
